# Evaluating the ability of end-point methods to predict the binding affinity tendency of protein kinase inhibitors†

**DOI:** 10.1039/d3ra04916g

**Published:** 2023-08-22

**Authors:** Martiniano Bello, Cindy Bandala

**Affiliations:** a Laboratorio de Diseño y Desarrollo de Nuevos Fármacos e Innovación Biotecnológica, Escuela Superior de Medicina, Instituto Politécnico Nacional, Plan de San Luis y Diaz Mirón s/n, Col. Casco de Santo Tomas Ciudad de México 11340 Mexico bellomartini@gmail.com mbellor@ipn.mx; b Escuela Superior de Medicina, Instituto Politécnico Nacional México City 11340 Mexico

## Abstract

Because of the high economic cost of exploring the experimental impact of mutations occurring in kinase proteins, computational approaches have been employed as alternative methods for evaluating the structural and energetic aspects of kinase mutations. Among the main computational methods used to explore the affinity linked to kinase mutations are docking procedures and molecular dynamics (MD) simulations combined with end-point methods or alchemical methods. Although it is known that end-point methods are not able to reproduce experimental binding free energy (Δ*G*) values, it is also true that they are able to discriminate between a better or a worse ligand through the estimation of Δ*G*. In this contribution, we selected ten wild-type and mutant cocrystallized EGFR–inhibitor complexes containing experimental binding affinities to evaluate whether MMGBSA or MMPBSA approaches can predict the differences in affinity between the wild type and mutants forming a complex with a similar inhibitor. Our results show that a long MD simulation (the last 50 ns of a 100 ns-long MD simulation) using the MMGBSA method without considering the entropic components reproduced the experimental affinity tendency with a Pearson correlation coefficient of 0.779 and an *R*^2^ value of 0.606. On the other hand, the correlation between theoretical and experimental ΔΔ*G* values indicates that the MMGBSA and MMPBSA methods are helpful for obtaining a good correlation using a short rather than a long simulation period.

## Abstract 2

Because of the high economic cost of exploring the experimental impact of mutations in kinase proteins, computational approaches have been employed as alternative methods for evaluating the structural and energetic aspects of kinase mutations.

## Graphical abstract

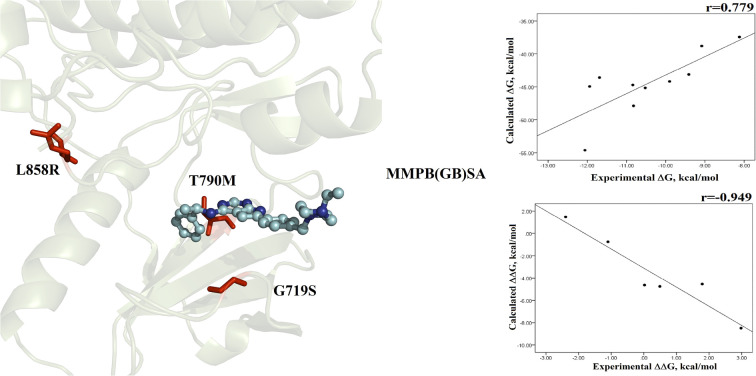

## 1. Introduction

**Fig. 1:**
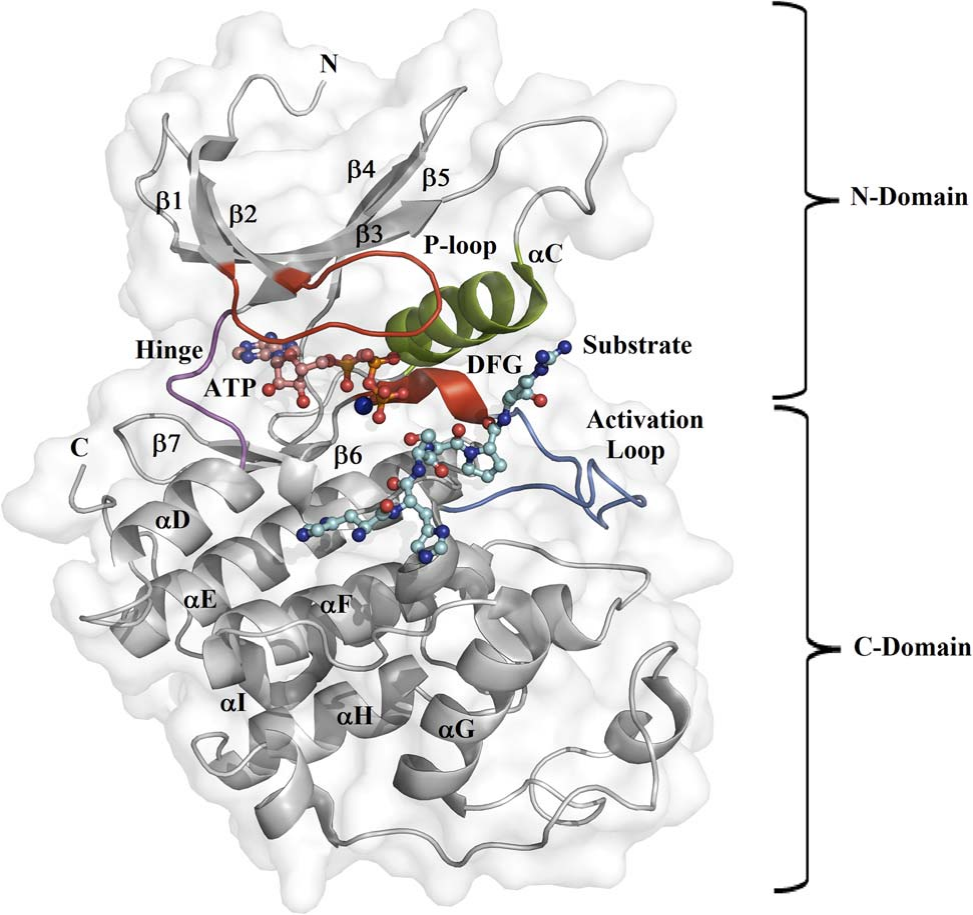
Design of the PK catalytic domain. The structural topology of a PK exemplified by CDK2 (PDB entry 1QMZ). The figure illustrates the N- and C-domains of CDK2, which forms a complex with ATP and the substrate at the catalytic binding domain. The activation loop is in blue, the P-loop in red, the αC-helix in green, the hinge region in magenta, the Mg^2+^ ion in orange, the ATP in pink, and the substrate in cyan.

Protein kinase (PK) enzymes are part of a huge superfamily and play an important role in diverse cellular activation events.^1^ PK enzymes catalyze the addition of a phosphate group to target residues (threonine, serine, tyrosine, and histidine) present in the catalytic site of enzymes, also known as the ATP binding site, and this represents a crucial process for the regulation of enzymatic activity. The three-dimensional structure of PK enzymes is canonically formed by two domains, also known as lobes, linked to each other through a flexible hinge region. The interface between these two domains forms a hydrophobic cleft that structures the ATP binding site (Fig. 1). The smaller N-terminal domain is structured by β-sheets (β1–β5) and one helix, known as αC, while the second C-terminal domain is enriched by several α-helices (αD–αI).^2–4^ PK enzymes share some regions involved in the catalytic process, that is, the activation loop, the DFG motif, the hinge, and the P-loop (Fig. 1). The catalytic activity of the enzymes is also connected with active or inactive states. In the active state, the activation loop is in the DFG-in state, whereas in the inactive state it is in the DFG-out conformation. In the inactive state, the Asp residue present in the DFG motif, which participates in the transfer of the phosphate group from ATP to the substrate, blocks the catalytic binding site avoiding substrate access, whereas in the active state, a conformational change in the activation loop allows access to the ATP binding site.^5,6^ Although normally the transition from the inactive to the active state is the result of phosphorylation at the activation loop, it can also be promoted by the binding of PK inhibitors.^7–9^

Phosphorylation of the enzyme contributes to modifying the physicochemical environment of the enzyme's active site, allowing PKs to develop their basic functions. However, some circumstances, such as changes in the expression level of PKs or mutations at the ATP binding site of PKs, affect diverse cellular events by modifying the catalytic activity of the enzyme or producing a constitutively active PK. Some mutations that affect the catalytic activity of the ATP binding site are mutations at the gatekeeper, the activation loop position, and the glycine-rich loop. L858R at the activation loop, G719S proximal to the glycine-rich loop in the epidermal growth factor receptor (EGFR), and T790M at the gatekeeper position are three examples of mutations that destabilize the inactive conformation.^10^ T790M in EGFR is an example of a gatekeeper mutation with bulkier side chain substitutions that block access to the catalytic binding site. This mutation impacts the hydrogen bonds between Thr and the ligand and also promotes the constitutive active state of EGFR,^11–13^ without impacting ATP binding.^14,15^

Recent advances in theoretical methods and the increase in experimental information about the impact of PK mutations on the inhibitor affinity of several PKs have allowed the development and evaluation of different methodologies for predicting the impact of mutations on inhibitor affinity. Although ideally it would be better to explore the impact of PK mutations through experimental methods, this turns out to be difficult because of time and economic aspects, so theoretical methods represent a good alternative for developing new PK inhibitors. Among the main theoretical approaches used to predict the difference in the binding affinity energy of wild-type *versus* mutated systems in protein–ligand systems are statistic-based machine-learning methods^16^ and structure-based methods.^17–19^ Of these two methods, structure-based methods are based on physical models. Although they are not as efficient and accurate as statistic-based machine-learning methods,^20,21^ structure-based methods have more advantages in the model implementation of different types of systems and in the interpretation of the results.^22–25^ Structure-based methods can be divided into alchemical and end-point methods. Alchemical methods, such as free energy perturbation (FEP) and thermodynamics integration (TI), are robust and precise methods for predicting binding free energies.^26^ However, these methods are computationally demanding and require long simulation times to converge, which makes them unsuitable for large-scale drug design projects. End-point methods, such as the molecular mechanics generalized-Born surface area (MMGBSA) and molecular mechanics Poisson–Boltzmann surface area (MMPBSA) methods,^27^ are the most employed methods for predicting the binding free energy (Δ*G*) and consider only the initial and final states of the evaluated system. MMGBSA and MMPBSA are computationally faster than alchemical methods and, because of that, have been successfully used in different types of systems and situations, such as getting insight into the drug resistance mechanisms of some pharmacological medicines.^25–31^ Comparing the MMGBSA and the FEP method on a massive number of mutations in protein–protein systems revealed better Pearson correlation coefficients against the experimental data for the FEP (*r* = 0.50–0.61) than for the MMGBSA (*r* = 0.14–0.18) method.^32^ On the other hand, Ikemura *et al.* determined the difference in binding free energy between PK inhibitors and EGFR containing rare mutations and compared the results against the experimental values using the MMGBSA and MMPBSA approaches, finding good Pearson correlation values (*r* = 0.57).^30^ More recently, Yu *et al.* predicted the binding free energy values for different kinds of protein–ligand systems containing mutations, using the MMGBSA and MMPBSA approaches but incorporating different computational strategies, such as different lengths of molecular dynamics (MD) simulations, different values of dielectric constants, and the incorporation of entropic effects in the final binding free energy.^33^ Based on this latter study, in which PK enzymes were not included, Yu *et al.* identified that 100 ns-long MD simulations benefit the prediction accuracy between theoretical and experimental ΔΔ*G* values of both methods with relatively good correlation values (*r* = 0.44). Furthermore, it was observed that the correlation is improved when the system contains multiple mutations. In this study, we predicted the binding free energy for a set of 10 wild-type and mutated EGFR–inhibitor complexes containing experimental affinity values, using the first 25 ns or the last 50 ns from 100 ns-long MD simulations combined with the MMGBSA and MMPBSA approaches with two dielectric constants and the incorporation of entropic effects in the binding free energy.

## 2. Methods

Table S1† shows our data set, containing single or double mutations within ten EGFR–inhibitor systems. For the preparation of the systems, all the crystal structures with missing regions were repaired using a Swiss model server.^34^ The EGFR–inhibitor complexes were constructed with antechamber and tleap modules present in the Amber22 package.^26,35^ The inhibitors were parametrized with the general Amber force field (GAFF)^26^ and AM1-BCC atomic charges,^36^ whereas the proteins were built using the Amber ff14SB force field.^37^ The systems were immersed in a truncated octahedral water box with a 12 Å TIP3P water model^38^ and neutralized by adding counterions to equilibrate the total charge.

### 2.1 Molecular dynamics (MD) simulations

With prior MD simulations, the systems were minimized and equilibrated, as follows. The minimization process consisted of 5000 cycles of steepest descent and 4000 cycles of conjugate gradient minimization. The temperature of the systems was increased for 200 ps from 0 to 310 K under an NVT ensemble using a Berendsen thermostat,^39^ during which heavy atoms in the protein were constrained with an elastic constant of 3 kcal mol^−1^ Å^−2^. Then, the density was equilibrated for 200 ps under an NTP ensemble using a Langevin thermostat^40,41^ and a Berendsen barostat,^39^ and a constant pressure was applied for 600 ps to accomplish equilibration at 310 K, maintaining the same constraints on the heavy atoms as used in the heating process. MD simulations were run under an NTP ensemble using a Langevin thermostat^40,41^ and a Berendsen barostat^39^ without any restrictions. The van der Waals and short-range electrostatic interactions were set to 10 Å, while the particle mesh Ewald (PME) algorithm^42^ was used to treat the long-range electrostatic interactions.^43^ The time step of the simulations was set to 2 fs, and the SHAKE algorithm^44^ was used to constrain the bond length between the hydrogen atoms and the linked heavy atoms. All the MD simulations were run in triplicate with the pmemd.cuda module in Amber22.^26,35^ A single joined trajectory was created by concatenating triplicate simulations and employed to estimate the root-mean-square deviation (RMSD) and the radius of gyration (RG) and to conduct the clustering analysis and binding free energy calculations.

### 2.2 Binding free energy studies with MMGBSA and MMPBSA

The binding free energy was determined for each system during the first 25 and the last 50 ns from the triplicate 100 ns-long MD trajectories, using the MMGBSA and MMPBSA methods and the single-method MD simulation protocol with the MMPBSA.py module^45^ present in the Amber22 simulation software. With these methods, the binding free energy can be decomposed into its energetic contributions, as follows:^26^Δ*G*_bind_ = *G*^complex^ − *G*^receptor^ − *G*^ligand^andΔ*G*_bind_ = Δ*E*_MM_ + Δ*G*_solvation_ − *T*Δ*S*where Δ*E*_MM_ contains the total gas-phase molecular mechanical energies of the molecular system, including five different terms: the bond (Δ*E*_bond_), the angle (Δ*E*_angle_), the dihedral (Δ*E*_dihedral_), the van der Waals (Δ*E*_vdw_), and the electrostatic (Δ*E*_ele_) energies. Δ*G*_solvation_ is the free energy penalty upon the molecular recognition process, and it is composed of polar (Δ*G*_PB/GB_) and nonpolar (Δ*G*_SA_) contributions. Δ*G*_PB/GB_ can be determined using the generalized-Born (GB) or the Poisson–Boltzmann (PB) model, whereas the nonpolar contribution to the solvation energy is determined using the solvent-accessible surface area (SASA) with the LCPO algorithm.^46^ In this contribution, Δ*G*_PB/GB_ was evaluated employing the GB model developed by Onufriev *et al.*^47^ (2004) and the PB model implemented by Tan *et al.*^48^ We explored two different interior dielectric constants (*ε*_in_ = 2 and 4) to compare the differences, since this constant has been reported to significantly impact the electrostatic contributions (Δ*E*_ele_ and Δ*G*_PB/GB_) of the binding free energy.^49,50^ −*T*Δ*S* is the result of the temperature (at 310 K) and the solute entropy arising from structural changes in the degrees of freedom of the water molecules. This was evaluated with 250 snapshots from the first 25 ns and the last 50 ns of the 100 ns-long MD simulations using the MMPBSA.py module.^45^ The binding free energy difference between the mutated and the wild-type systems was stated (ΔΔ*G* = Δ*G*_MT_ − Δ*G*_WT_).

### 2.3 Molecular docking

Docking studies were performed using two different algorithms: MOE 2014.09,^51^ and SwissDock.^52^ The ligand geometries were minimized with Avogadro^53^ with a UFF force field, using the steepest descent method followed by conjugate gradient algorithms. Docking calculations with MOE 2014.09 were carried out using the triangle matcher function to create the initial binding poses. The best 30 poses from the London dG score were then rescored using GBVI/WSA dG. For docking studies with SwissDock, the default parameters were used. For all the cases, the search space was set at the known binding pocket, and the receptor–ligand system with the lowest binding score was chosen as the representative complex.

## 3. Results and discussion

**Fig. 2:**
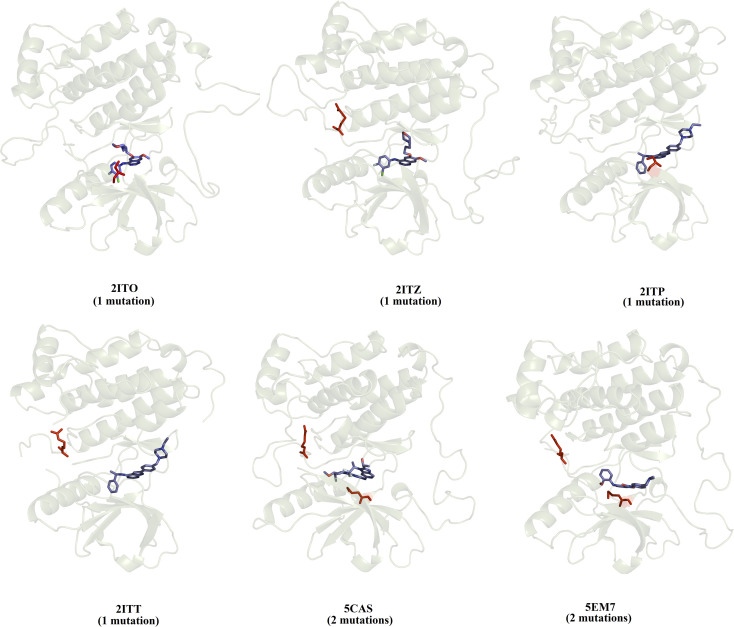
Structural details of the investigated systems. The set of mutated EGFR–inhibitor cocrystallized complexes.

We selected a set of 10 cocrystallized wild-type and mutated EGFR–inhibitor complexes containing experimental affinity values (Table S1†). Fig. 2 illustrates six of these mutated EGFR–inhibitor cocrystallized complexes. Of the evaluated systems, four contain a single mutation, and two have two mutations almost all evaluated systems showed that the mutations are located close to the cocrystallized ligand, suggesting essential interactions between the ligand and the ligand binding site. These systems were submitted to MD simulations combined with various protocols of end-point binding free energy methods with the purpose of evaluating the ability of this methodology to predict the impact of mutations on the binding affinity. We also evaluate the binding affinity of these complexes using two different docking algorithms, the results of which are compared with the MD simulation results.

### 3.1 Stabilization of the wild-type and mutated EGFR–inhibitor complexes

The RMSD and RG plots of the EGFR–inhibitor complexes illustrate that all the complexes reached equilibrated values between 20 and 50 ns, with average RMSD values between 2.05 and 5.52 Å, whereas that average RG values fluctuated between 19.9 and 20.8 Å (Table S2†). Based on this analysis, a clustering analysis was performed using both the equilibrated simulation time (the last 50 ns) and the time before reaching the equilibrium (the first 25 ns) to obtain the most populated complexes and evaluate the RMSD value of the ligand binding pose obtained during simulations concerning the cocrystallized ligand conformation. This analysis showed that the ligand poses exhibited RMSD values lower than 2.0 for the MD simulation studies before and after reaching equilibrium (Table S3†). The results indicated similar ligand displacement with respect to the cocrystallized ligand conformer before and after reaching equilibrium, except for the 2j6m system, which improves its RMSD value during the last 50 ns.

### 3.2 Impact of the MD simulation time on the binding free energy

The minimized wild-type and mutated cocrystallized PK–ligand complexes were submitted to MD simulations combined with MMGBSA and MMPBSA using a dielectric constant (*ε*_in_ = 2) that has been reported to be the most suitable one for studying protein–ligand systems.^49,50,54,55^ In addition, we found similar results with a higher dielectric contact (*ε*_in_ = 4; data not shown), as previously reported,^33^ so in this research, we showed only results for *ε*_in_ = 2.

**Fig. 3:**
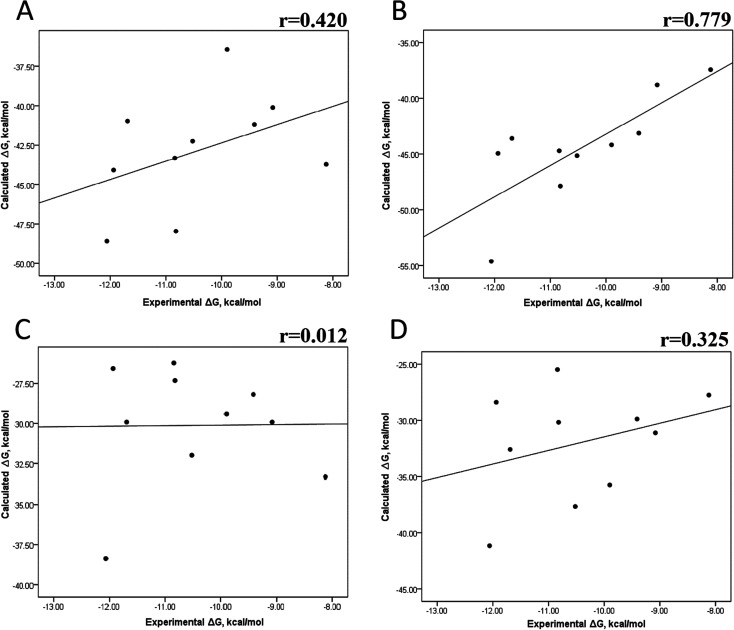
Effect of MD simulation time on the binding free energy using the MMGBSA and MMPBSA methods. The Δ*G* values determined using the MMGBSA approach, considering the first 25 ns (A) and the last 50 ns (B) of a 100 ns-long MD simulation. The Δ*G* values determined using the MMPBSA approach, considering the first 25 ns (C) and the last 50 ns (D) of a 100 ns-long MD simulation.

**Fig. 4:**
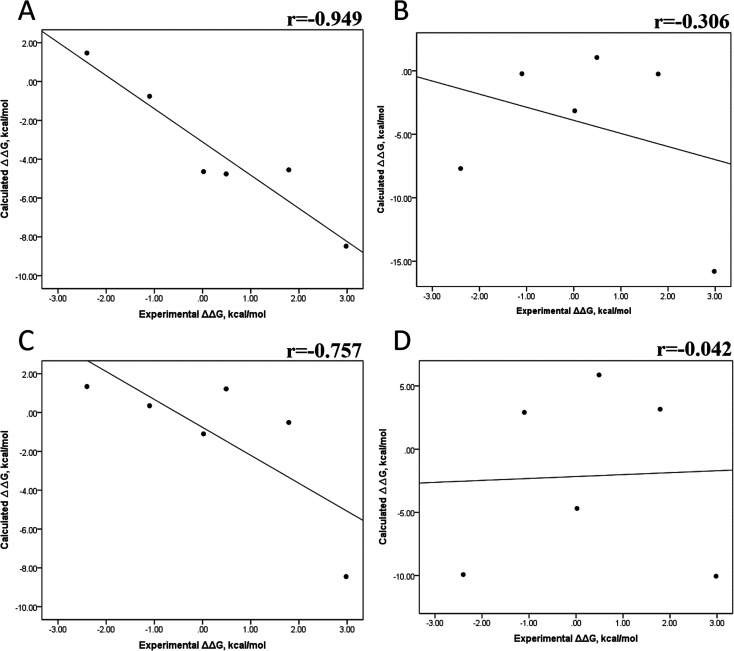
Effect of MD simulation time on the relative binding free energy using the MMGBSA and MMPBSA methods. The calculated ΔΔ*G* values between the mutated and the wild-type systems determined using the MMGBSA approach, considering the first 25 ns (A) and the last 50 ns (B) of a 100 ns-long MD simulation. The calculated ΔΔ*G* values determined using the MMPBSA approach, considering the first 25 ns (C) and the last 50 ns (D) of a 100 ns-long MD simulation.

Tables S4–S7 and S12† show all the binding free energy (Δ*G*) values using MMGBSA (Δ*G*_gb_) or MMPBSA (Δ*G*_pb_) during the first 25 ns or the last 50 ns of the 100 ns-long MD simulations. The Pearson correlation coefficients between the calculated and the experimental Δ*G* values showed a better correlation for the last 50 ns (*r* = 0.779) (Fig. 3B) than for the first 25 ns (*r* = 0.420) (Fig. 3A). The Δ*G*_pb_ values obtained with the MMPBSA approach showed a lack of correlation for the first 25 ns (*r* = 0.012) (Fig. 3C), which improved for the last 50 ns (*r* = 0.325) (Fig. 3D). Although a comparison between the predicted and experimental Δ*G* values (Tables S1 and S12†) clearly showed that the MMGBSA or MMPBSA methods were not able to reproduce the experimental Δ*G* values, our results indicated that the MMGBSA method reproduced the affinity tendency for the group of wild-type and mutant EGFR–inhibitor complexes with a coefficient value similar to that previously reported for different protein–ligand systems^33,56^ and between some PK inhibitors and EGFR containing rare mutations.^30^ Based on this result, we also determined the correlation between the mutated and the wild-type systems (ΔΔ*G* = Δ*G*_MT_ − Δ*G*_WT_). Fig. 4A and B for MMGBSA show that with an increase in the MD simulation time, the Pearson correlation coefficient decreases from −0.949 of the conformers during the first 25 ns to −0.306 during the last 50 ns of the 100 ns-long MD simulations. A similar trend is shown for the results using the MMPBSA approach. In this case, the Pearson correlation coefficient also reduces from −0.757 of the conformers during the first 25 ns to −0.042 during the last 50 ns of the 100 ns-long MD simulations (Fig. 4C and D). A comparison of the correlations of calculated Δ*G* (Fig. 3) or ΔΔ*G* (Fig. 4) using the two methods reveals that the better method for reproducing the experimental Δ*G* tendency for EGFR–inhibitor systems is the MMGBSA method using more extended MD simulations. On the other hand, the predicted *versus* experimental ΔΔ*G* correlation indicates that the MMGBSA and MMPBSA methods help obtain good correlations when short rather than long simulation periods are used.

### 3.3 Impact of the conformational entropy on the binding affinity

**Fig. 5:**
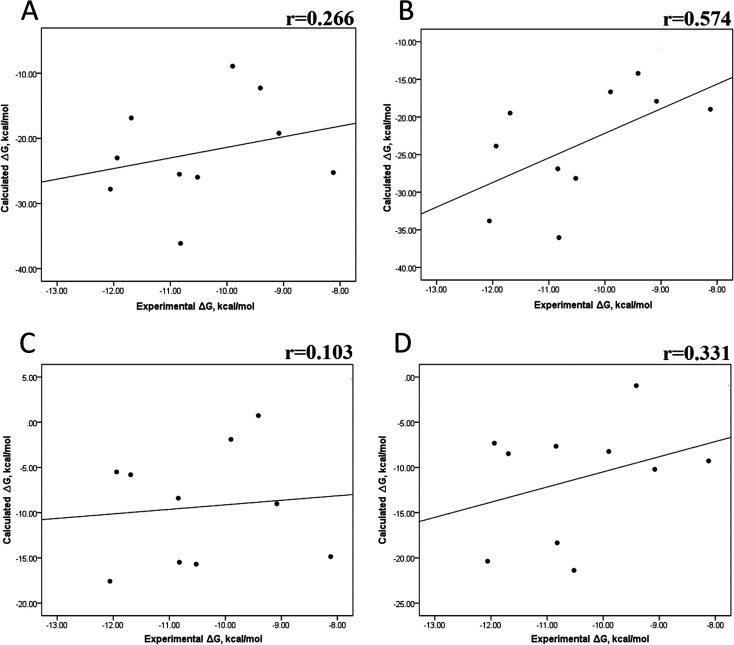
Impact of MD simulation time on the binding free energy using the MMGBSA and MMPBSA methods, considering the entropic component. Δ*G* values determined using the MMGBSA approach, considering the first 25 ns (A) and the last 50 ns (B) of a 100 ns-long MD simulation. Δ*G* values determined using the MMPBSA approach, considering the first 25 ns (C) and the last 50 ns (D) of a 100 ns-long MD simulation.

**Fig. 6:**
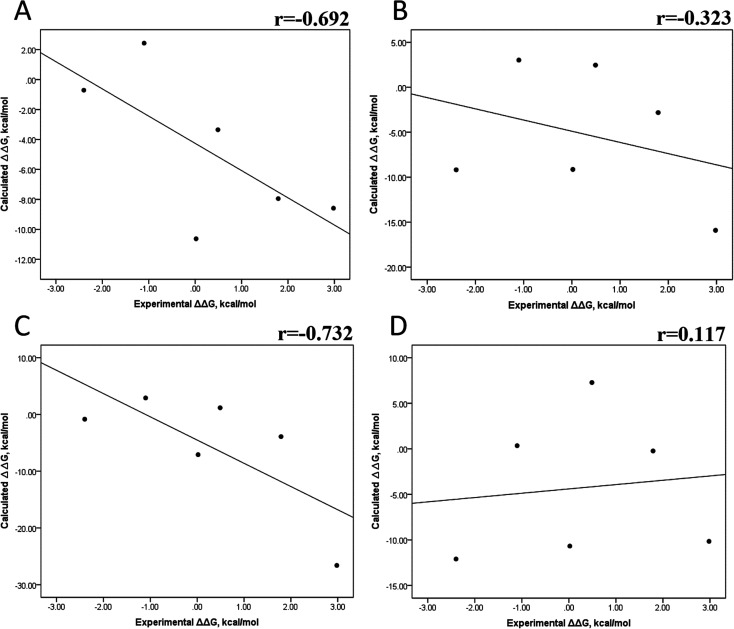
Impact of MD simulation time on the absolute binding free energy using the MMGBSA and MMPBSA methods. Calculated *versus* experimental ΔΔ*G* between the mutated and the wild-type systems determined using the MMGBSA approach, considering the first 25 ns (A) and the last 50 ns (B) of a 100 ns-long MD simulation and considering the entropic contribution. Calculated *versus* experimental ΔΔ*G* between the mutated and the wild-type systems using the MMPBSA approach, considering the first 25 ns (C) and the last 50 ns (D) of a 100 ns-long MD simulation.

We also evaluated the impact of the conformational entropy, although it has been stated that introducing the entropy contribution in the prediction of the binding free energy is not able to improve the correlation between the predicted and the experimental binding free energy.^54^ Tables S8–S11 and S12† also present all the Δ*G* values using the MMGBSA or MMPBSA method, considering the entropic component. The correlation between the calculated and experimental Δ*G* values with MMGBSA during the first 25 ns (Fig. 5A) and the last 50 ns (Fig. 5B) of the 100 ns-long MD simulations revealed a better correlation for the last 50 ns (*r* = 0.574) than for the first 25 ns (*r* = 0.266). The correlation obtained using the MMPBSA approach was worse than that obtained using the MMGBSA approach, in which a higher correlation was observed during the last 50 ns (*r* = 0.331) than during the first 25 ns (*r* = 0.103). The ΔΔ*G* values were also calculated from our Δ*G* values for estimating the correlation between the predicted and experimental ΔΔ*G* values (Fig. 6).

In panels A and B of Fig. 6, a decrease in the Pearson correlation coefficient from −0.692 during the first 25 ns to −0.323 during the last 50 ns is observed for the MMPBSA approach. Similarly, for the MMPBSA approach, it was observed that the Pearson correlation coefficient reduces from −0.732 during the first 25 ns to 0.117 during the last 50 ns of the 100 ns-long MD simulations.

A comparison of the correlations of Δ*G* (Fig. 5) or ΔΔ*G* (Fig. 6) using the two methods reveals that the best method for reproducing the experimental Δ*G* tendency in EGFR–inhibitor systems is also the MMGBSA method using long MD simulation periods, as observed for the Δ*G* values without considering the entropic component (Fig. 3). However, it was also observed that incorporating entropy into the prediction seriously weakens the prediction accuracy, as previously reported.^33,54^ On the other hand, the predicted *versus* experimental ΔΔ*G* analysis indicated that short MD simulations generate a better correlation value for both methods than long MD simulations when the entropic component is incorporated in Δ*G* prediction. This result points out that mutations near the binding site do not require to experience significant conformational changes through long MD simulations to reach good theoretical *versus* experimental ΔΔ*G* tendency, contrasting with long-distance mutations of other protein systems that require long MD simulations to get good theoretical *versus* experimental ΔΔ*G* tendencies.^33^

### 3.4 Evaluating the ability of docking programs to predict the affinity

**Fig. 7:**
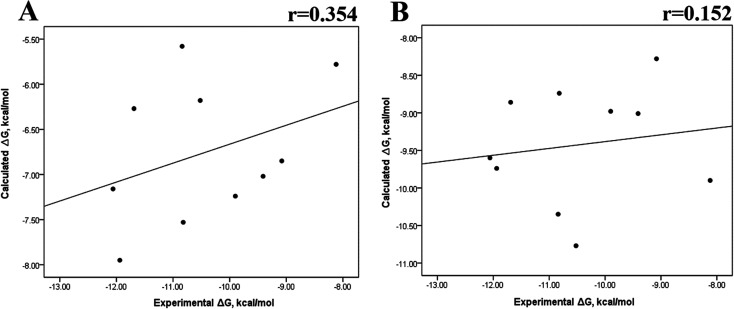
Calculated *versus* experimental Δ*G* between the mutated and the wild-type systems obtained using docking methods. Correlation obtained using MOE (A), and SwissDock (B).

**Fig. 8:**
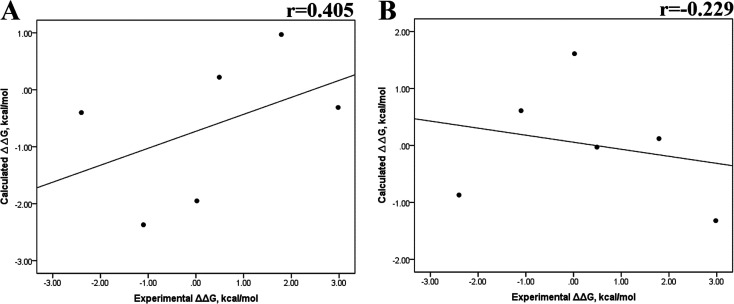
Calculated *versus* experimental ΔΔ*G* between the mutated and the wild-type systems obtained using docking methods. Correlation obtained using MOE (A), and SwissDock (B).

Previous research evaluated the ability of six different docking programs—four web-based knowledge-based scoring functions (DSX-ONLINE, KDEEP, Pose&Rank, and PRODIGY-LIG) and two general scoring functions (HADDOCK2.2 and PDBePISA)—to determine Δ*G*_docking_ for several wild-type and their respective mutant kinases. Correlation studies between calculated and experimental ΔΔ*G* values (IC_50_, Kd, Ki) showed they could predict whether a mutation improves or worsens the binding affinity. Interestingly, the best Pearson correlation was observed for the web-based protein–ligand scoring function DSX-ONLINE with the Ki data set.^57^ Because we observed that the best correlation between theoretical and experimental Δ*G* values was obtained for the last 50 ns-long MD simulations (*r* = 0.779) without considering the entropic contributions, suggesting that the conformational changes did not greatly impact the theoretical Δ*G* values, we performed docking simulations using two different general scoring functions to obtain the predicted Δ*G* values (Table S13†) and evaluate their correlation with experimental Δ*G* values. RMSD analysis between the ligand binding pose generated by docking procedures and the cocrystallized ligand reveals RMSD values lower than 2.5 Å, however, these values are in general higher than that of MD simulations (Table S3†). A comparison of RMSD values of docking with MD simulations indicates that the ligand experiment shows lower displacement with respect to the cocrystallized ligand conformer than docking procedures, suggesting that docking procedures may unfavorably impact the protein–ligand interactions with respect to MD simulations. Fig. 7 showed a poor Pearson correlation coefficient of 0.354 for MOE (Fig. 7A), and 0.152 for SwissDock (Fig. 7B), respectively. The value reached by these two docking programs indicates that they are worse than MMGBSA and MMPBSA methods for reproducing the experimental Δ*G* tendency. The ΔΔ*G* values were also calculated from our docking Δ*G* values for estimating the correlation between the calculated and experimental ΔΔ*G* values (Fig. 8). In Fig. 8, a Pearson correlation coefficient of 0.405 is observed for MOE (Fig. 8B) and of −0.229 for SwissDock (Fig. 8C). A comparison between the two docking algorithms indicates that despite the low capacity of both algorithms for generating good correlation values, MOE shows a better performance than SwissDock.

## 4. Conclusion

In this research, we explore the performance of the MMGBSA and MMPBSA methods under different conditions to evaluate their ability to reproduce the experimental binding tendency and the difference between wild-type and mutant EGFR–inhibitor systems. In addition, we compare these two methodologies with two docking algorithms. Our results indicate that the MMGBSA protocol without the entropic component and with a long MD simulation time of at least 100 ns is useful for obtaining good reproducibility of the experimental Δ*G* tendency, indicating that some conformational changes are needed to reproduce the Δ*G* tendency. On the other hand, the ΔΔ*G* values between wild-type and mutant systems showed a good reproducibility tendency for both the MMGBSA and the MMPBSA protocol without the entropic component and with short MD simulations of about 25 ns. The results indicate that mutations close to the binding site do not require conformational rearrangement to reach a good experimental ΔΔ*G* tendency. On the other hand, the two docking programs explored failed to reproduce experimental Δ*G* and ΔΔ*G* tendencies, corroborating the necessity of MMGBSA or MMPBSA methods to reproduce experimental tendencies.

## Data availability

The datasets supporting the conclusions of this research are contained within the paper and its additional files.

## Conflicts of interest

The authors declare they have no conflict of interest in terms of the content of this manuscript.

## Supplementary Material

RA-013-D3RA04916G-s001

## References

[cit1] Manning G. and Hunter T., Eukaryotic Kinomes: Genomics and Evolution of Protein Kinases, in Handb. Cell Signaling, Academic Press, California, 2nd edn, 2010, pp. 393–397

[cit2] Bossemeyer D. (1995). Protein kinases—structure and function. FEBS Lett..

[cit3] Kornev A.-P., Taylor S. S. (2015). Dynamics-driven allostery in protein kinases. Trends Biochem. Sci..

[cit4] Weber T. J. and Qian W., Protein Kinases, in Compr. Toxicol, Elsevier Ltd, AL, USA, 3rd edn, 2018, pp. 264–285

[cit5] Cruzalegui F. (2010). Protein Kinases: From Targets to Anti-Cancer Drugs. Ann. Pharm. Fr..

[cit6] Huse M., Kuriyan J. (2002). The Conformational Plasticity of Protein Kinases. Cell.

[cit7] Adams J. A. (2003). Activation Loop Phosphorylation and Catalysis in Protein Kinases: Is There Functional Evidence for the Autoinhibitor Model?. Biochemistry.

[cit8] Martin M.-P., Zhu J.-Y., Lawrence H. R., Pireddu R., Luo Y., Alam R., Ozcan S., Sebti S. M., Lawrence N. J., Schönbrunn E. (2012). A Novel Mechanism by Which Small Molecule Inhibitors Induce the DFG Flip in Aurora A. ACS Chem. Biol..

[cit9] Vijayan R. S. K., He P., Modi V., Duong-Ly K. C., Ma H., Peterson J. R., Dunbrack R. L., Levy R. M. (2015). Conformational Analysis of the DFG-Out Kinase Motif and Biochemical Profiling of Structurally Validated Type II Inhibitors. J. Med. Chem..

[cit10] Torkamani A., Verkhivker G., Schork N. J. (2009). Cancer Driver Mutations in Protein Kinase Genes. Cancer Lett..

[cit11] Brognard J., Hunter T. (2011). Protein Kinase Signaling Networks in Cancer. Curr. Opin. Genet. Dev..

[cit12] Mortlock A. , Foote K., Kettle J. and Aquila B., Kinase Inhibitors in Cancer, in Ref. Module Chem., Mol. Sci. Chem. Eng., Elsevier Ltd, 2014, p. B9780124095472110000

[cit13] Mortlock A. A. , Wilson D. M., Kettle J. G., Goldberg F. W. and Foote K. M., Selective Kinase Inhibitors in Cancer, in Compr. Med. Chem. III, Elsevier Ltd, 2017, pp. 39–75

[cit14] Daub H., Specht K., Ullrich A. (2004). Strategies to Overcome Resistance to Targeted Protein Kinase Inhibitors. Nat. Rev. Drug Discovery.

[cit15] Barouch-Bentov R., Sauer K. (2011). Mechanisms of Drug Resistance in Kinases. Expert Opin. Investig. Drugs.

[cit16] Koohi-Moghadam M., Wang H., Wang Y., Yang X. (2019). Predicting disease associated mutation of metal-binding sites in proteins using a deep learning approach. Nat. Mach. Intell..

[cit17] Hauser K., Negron C., Albanese S. K., Ray S., Steinbrecher T., Abel R., Chodera J. D., Wang L. (2018). Predicting Resistance of Clinical Abl Mutations to Targeted Kinase Inhibitors Using Alchemical Free-Energy Calculations. Commun. Biol..

[cit18] Aldeghi M., Gapsys V., de Groot B. L. (2019). Predicting kinase inhibitor resistance: physics-based and data-driven approaches. ACS Cent. Sci..

[cit19] Bhati A. P., Wan S., Coveney P. V. (2019). Ensemble-based replica exchange alchemical free energy methods: the effect of protein mutations on inhibitor binding. J. Chem. Theory Comput..

[cit20] Iqbal S., Li F., Akutsu T., Ascher D. B., Webb G. I., Song J. (2021). Assessing the performance of computational predictors for estimating protein stability changes upon missense mutations. Briefings Bioinf..

[cit21] Li B., Yang Y. T., Capra J. A., Gerstein M.-B. (2020). Predicting changes in protein thermodynamic stability upon point mutation with deep 3D convolutional neural networks. PLoS Comput. Biol..

[cit22] Gapsys V., Pérez-Benito L., Aldeghi M., Seeliger D., van Vlijmen H., Tresadern G., de Groot B. L. (2019). Large scale relative protein ligand binding afnities using non-equilibrium alchemy. Chem. Sci..

[cit23] Jespers W., Isaksen G. V., Andberg T. A., Vasile S., van Veen A., Åqvist J., Bramdsdal B. O., Gutiérrez-de-Terán H. (2019). QresFEP: an automated protocol for free energy calculations of protein mutations in Q. J. Chem. Theory Comput..

[cit24] Li Z., Huang Y., Wu Y., Chen J., Wu D., Zhan C., Luo H. (2019). Absolute binding free energy calculation and design of a subnanomolar inhibitor of phosphodiesterase-10. J. Med. Chem..

[cit25] Chen J., Wang X., Pang L., Zhang J. Z. H., Zhu T. (2019). Efect of mutations on binding of ligands to guanine riboswitch probed by free energy perturbation and molecular dynamics simulations. Nucleic Acids Res..

[cit26] Wang E., Sun H., Wang J., Wang Z., Liu H., Zhang J. Z. H., Hou T. (2019). End-point binding free energy calculation with MM/PBSA and MM/GBSA: strategies and applications in drug design. Chem. Rev..

[cit27] Kollman P. A., Massova I., Reyes C., Kuhn B., Huo S., Chong L., Lee M., Lee T., Duan Y., Wang W., Donini O., Cieplak P., Srinivasan J., Case D. A., Cheatham 3rd T. E. (2000). Calculating structures and free energies of complex molecules: combining molecular mechanics and continuum models. Acc. Chem. Res..

[cit28] Kong X., Sun H., Pan P., Tian S., Li D., Li Y., Hou T. (2016). Molecular principle of the cyclindependent kinase selectivity of 4-(thiazol-5-yl)-2-(phenylamino) pyrimidine-5-carbonitrile derivatives revealed by molecular modeling studies. Phys. Chem. Chem. Phys..

[cit29] Sun H., Li Y., Li D., Hou T. (2013). Insight into crizotinib resistance mechanisms caused by three mutations in ALK tyrosine kinase using free energy calculation approaches. J. Chem. Inf. Model..

[cit30] Ikemura S., Yasuda H., Matsumoto S., Kamada M., Hamamoto J., Masuzawa K., Kobayashi K., Manabe T., Arai D., Nakachi I., Kagawa I., Ishioka K., Nakamura M., Namkoong H., Naoki K., Ono F., Araki M., Kanada R., Ma B., Hayashi Y., Mimaki S., Yoh K., Kobayashi S. S., Kohno T., Okuno Y., Goto K., Tsuchihara K., Soejima K. (2019). Molecular dynamics simulation-guided drug sensitivity prediction for lung cancer with rare EGFR mutations. Proc. Natl. Acad. Sci. U.S.A..

[cit31] Fulle S., Saini J. S., Homeyer N., Gohlke H. (2015). Complex long-distance efects of mutations that confer linezolid resistance in the large ribosomal subunit. Nucleic Acids Res..

[cit32] Clark A. J., Negron C., Hauser K., Sun M., Wang L., Abel R., Friesner R. A. (2019). Relative binding afnity prediction of charge-changing sequence mutations with FEP in protein–protein interfaces. J. Mol. Biol..

[cit33] Yu Y., Wang Z., Wang L., Tian S., Hou T., Sun H. (2022). Predicting the mutation effects of protein-ligand interactions via end-point binding free energy calculations: strategies and analyses. J. Cheminf..

[cit34] Waterhouse A., Bertoni M., Bienert S., Studer G., Tauriello G., Gumienny R., Heer F. T., de Beer T. A. P., Rempfer C., Bordoli L., Lepore R., Schwede T. (2018). SWISS-MODEL: homology modelling of protein structures and complexes. Nucleic Acids Res..

[cit35] Case D. A., Cheatham 3rd T. E., Darden T., Gohlke H., Luo R., Merz Jr K. M., Onufriev A., Simmerling C., Wang B., Woods R. J. (2005). The Amber biomolecular simulation programs. J. Comput. Chem..

[cit36] Jakalian A., Jack D. B., Bayly C. I. (2002). Fast, efficient generation of high-quality atomic charges. AM1-BCC model: II. Parameterization and validation. J. Comput. Chem..

[cit37] Tian C., Kasavajhala K., Belfon K. A., Raguette L., Huang H., Migues A. N., Bickel J., Wang Y., Pincay J., Wu Q., Simmerling C. (2020). ff19SB: Amino-acid-specific protein backbone parameters trained against quantum mechanics energy surfaces in solution. J. Chem. Theory Comput..

[cit38] Jorgensen W. L., Chandrasekhar J., Madura J. D., Impey R. W., Klein M. L. (1983). Comparison of simple potential functions for simulating liquid water. J. Chem. Phys..

[cit39] Berendsen H. J. C., Postma J. P. M., van Gunsteren W. F., DiNola A., Haak J. R. (1984). Molecular dynamics with coupling to an external bath. J. Chem. Phys..

[cit40] Uberuaga B. P., Anghel M., Voter A. F. (2004). Synchronization of trajectories in canonical molecular-dynamics simulations: Observation, explanation, and exploitation. J. Chem. Phys..

[cit41] Sindhikara D. J., Kim S., Voter A. F., Roitberg A. E. (2009). Bad Seeds Sprout Perilous Dynamics: Stochastic Thermostat Induced Trajectory Synchronization in Biomolecules. J. Chem. Theory Comput..

[cit42] Darden T., York D., Pedersen L. (1993). Particle mesh Ewald: AnN·log(N) method for Ewald sums in large systems. J. Chem. Phys..

[cit43] York D. M., Darden T. A., Pedersen L. G. (1993). The effect of long-range electrostatic interactions in simulations of macromolecular crystals: a comparison of the Ewald and truncated list methods. J. Chem. Phys..

[cit44] Ryckaert J. P., Ciccotti G., Berendsen H. J. C. (1997). Numerical integration of the Cartesian equations of motion of a system with constraints: molecular dynamics of n-alkanes. J. Comput. Phys..

[cit45] Miller 3rd B. R., McGee T. D., Swails J. M., Homeyer N., Gohlke H., Roitberg A. E. (2012). MMPBSA.py: an efficient program for end-state free energy calculations. J. Chem. Theory Comput..

[cit46] Weiser J., Shenkin P. S., Still W.-C. (1999). Approximate atomic surfaces from linear combinations of pairwise overlaps (LCPO). J. Comput. Chem..

[cit47] Onufriev A., Bashford D., Case D.A. (2004). Exploring protein native states and large-scale conformational changes with a modified generalized born model. Proteins.

[cit48] Tan C., Yang L., Luo R. (2006). How well does Poisson-Boltzmann implicit solvent agree with explicit solvent? A quantitative analysis. J. Phys. Chem. B.

[cit49] Sun H., Li Y., Tian S., Xu L., Hou T. (2014). Assessing the performance of MM/PBSA and MM/GBSA methods. 4. Accuracies of MM/PBSA and MM/GBSA methodologies evaluated by various simulation protocols using PDBbind data set. Phys. Chem. Chem. Phys..

[cit50] Sun H., Duan L., Chen F., Liu H., Wang Z., Pan P., Xhu F., Zhang J. Z. H., Hou T. (2018). Assessing the performance of MM/PBSA and MM/GBSA methods. 7. Entropy efects on the performance of end-point binding free energy calculation approaches. Phys. Chem. Chem. Phys..

[cit51] Molecular Operating Environment (MOE) , Chemical Computing Group Inc. Home Page, Montreal, Quebec, Canada, 2014, https://www.chemcomp.com/

[cit52] Grosdidier A., Zoete V., Michielin O. (2011). SwissDock, a protein-small molecule docking web service based on EADock DSS. Nucleic Acids Res..

[cit53] Hanwell M. D., Curtis D. E., Lonie D. C., Vandermeersch T., Zurek E., Hutchison G. R. (2012). Avogadro: an advanced semantic chemical editor, visualization, and analysis platform. J. Cheminf..

[cit54] Soderhjelm P., Kongsted J., Ryde U. (2010). Ligand affinities estimated by quantum chemical calculations. J. Chem. Theory Comput..

[cit55] Genheden S., Ryde U. (2015). The MM/PBSA and MM/GBSA methods to estimate ligand-binding affinities. Expert Opin. Drug Discovery.

[cit56] Hou T., Wang J., Li Y., Wang W. (2011). Assessing the performance of the molecular mechanics/Poisson Boltzmann surface area and molecular mechanics/generalized Born surface area methods. II. The accuracy of ranking poses generated from docking. J. Comput. Chem..

[cit57] Erguven M., Karakulak T., Diril M. K., Karaca E. (2021). How Far Are We from the Rapid Prediction of Drug Resistance Arising Due to Kinase Mutations?. ACS Omega.

