# Supplementary material for: Evaluating the ability of end-point methods to predict the binding affinity tendency of protein kinase inhibitors
Source: RSC Adv. 2023 Aug 22;13(36):25118–28. doi: 10.1039/d3ra04916g (PMC10443623; doi:10.1039/d3ra04916g)
Supplement: RA-013-D3RA04916G-s001 [file RA-013-D3RA04916G-s001.pdf]

Electronic Supplementary Material (ESI) for RSC Advances.
This journal is © The Royal Society of Chemistry 2023

Supplementary Material

Table S1. The PDB entries of the evaluated systems, protein names, mutation remarks, the experimental binding affinity data, and references.

PDB entry	Protein Kinase	Ligand	Remarks	Missing regions close to the binding site	ΔG_{exp} (kcal/mol)	References
2ity	EGFR	IRE	WT	866-875	-9.90	[1]
2ito	EGFR	IRE	MUT (G719S)	867-875	-9.41	[1]
2itz	EGFR	IRE	MUT (L858R)	866-875	-11.69	[1]
2j6m	EGFR	AEE	WT	No	-10.84	[1]
2itp	EGFR	AEE	MUT (G719S)	No	-10.82	[1]
2itt	EGFR	AEE	MUT (L858R)	865-866	-11.94	[1]
5cav	EGFR	4ZQ	WT	No	-9.08	[2]
5cas	EGFR	4ZQ	MUT (T790M, L858R)	999-1006	-12.06	[2]
5em8	EGFR	5Q4	WT	747-749	-8.12	[3]
5em7	EGFR	5Q4	MUT (T790M, L858R)	747-749	-10.52	[3]

ΔG_{exp} values were determined using experimental Ki and Kd values for each system present in references.

Table S2. Average RMSD and Rg values of the EGRF-inhibitor systems.

Systems	RMSD	Rg
2ity	3.25 ± 0.21	20.72 ± 0.14
2ito	3.50 ± 0.19	20.70 ± 0.12
2itz	3.12 ± 0.25	20.82 ± 0.11
2j6m	2.05 ± 0.20	20.04 ± 0.09
2itp	2.32 ± 0.38	19.99± 0.20
2itt	2.08 ± 0.27	19.99 ± 0.12
5cav	4.78 ± 0.21	20.70 ± 0.14
5cas	4.95 ± 0.35	20.89 ± 0.09
5em8	4.32 ± 0.56	20.40 ± 0.15
5em7	5.52 ± 0.55	22.23 ± 0.21

Table S3. RMSD of the cocrystallized ligand into the protein-ligand systems.

Systems	$RMSD_{MD\text{-}first\text{-}25ns}$	$RMSD_{MD\text{-}last\text{-}50ns}$	$RMSD_{MOE}$	$RMSD_{SWDOCK}$
2ity	0.99	0.95	2.40	2.50
2ito	1.01	1.29	1.70	2.50
2itz	0.83	0.97	1.10	1.00
2j6m	1.93	0.88	2.30	0.60
2itp	0.71	1.01	2.45	2.00
2itt	0.49	0.90	1.36	1.10
5cav	1.01	1.34	1.40	2.50
5cas	0.50	0.95	1.36	0.60
5em8	1.12	0.95	1.90	1.40
5em7	1.23	1.18	2.00	1.80

This table describe the RMSD (Å) of the cocrystallized ligand during the first 25 ($RMSD_{MD\text{-}first\text{-}25ns}$) and the last 50 ns ($RMSD_{MD\text{-}last\text{-}50ns}$) of the 100-ns-long MD simulations, as well as the RMSD of docking calculations using MOE ($RMSD_{MOE}$) or SWISSDOCK ($RMSD_{SWDOCK}$).

Table S4. Binding free energy values for the protein-ligand systems during the first 25ns ($\Delta G_{gb\text{-}25ns}$) using the MMGBSA approach (in units of kcal/mol).

System	ΔE_{vdw}	ΔE_{ele}	$\Delta G_{ele,sol}$	$\Delta G_{npol,sol}$	$\Delta G_{gb\text{-}25ns}$
2ity	-45.03 ± 3.7	-139.79 ± 25.5	154.14 ± 23.4	-5.75 ± 0.63	-36.43 ± 4.6
2ito	-50.31 ± 3.3	-136.72 ± 16.3	152.29 ± 16.4	-6.44 ± 0.43	-41.19 ± 3.1
2itz	-51.04 ± 3.4	-136.49 ± 14.7	153.03 ± 13.6	-6.47 ± 0.43	-40.98 ± 4.0
2j6m	-50.14 ± 3.0	24.36 ± 4.4	-11.41 ± 5.0	-6.13 ± 0.28	-43.32 ± 3.5
2itp	-52.34 ± 3.3	16.11 ± 16.3	-5.34 ± 1.2	-6.39 ± 0.40	-47.96 ± 3.3
2itt	-49.30 ± 2.7	18.93 ± 11.3	-7.65 ± 1.9	-5.99 ± 0.37	-44.08 ± 3.1
5cav	-51.51 ± 2.5	-34.75 ± 6.2	52.37 ± 5.0	-6.21 ± 0.23	-40.11 ± 3.9
5cas	-50.54 ± 3.1	-39.48 ± 6.0	47.89 ± 5.2	-6.46 ± 0.25	-48.59 ± 5.0
5em8	-49.02 ± 4.3	-147.59 ± 18.9	159.17 ± 19.7	-6.27 ± 0.40	-43.72 ± 4.7
5em7	-49.22 ± 3.3	-131.16 ± 19.7	144.23 ± 19.5	-6.10 ± 0.33	-42.25 ± 3.6

Table S5. Binding free energy values for the protein-ligand systems during last 50 ns ($\Delta G_{gb\text{-}50ns}$) using the MMGBSA approach (in units of kcal/mol).

Systems	ΔE_{vdw}	ΔE_{ele}	$\Delta G_{ele,sol}$	$\Delta G_{npol,sol}$	$\Delta G_{gb\text{-}50ns}$
2ity	-48.02 ± 3.0	-162.68 ± 17.0	172.83 ± 15.9	-6.30 ± 0.38	-44.18 ± 4.3
2ito	-50.50 ± 3.7	-154.00 ± 21.1	168.00 ± 18.5	-6.63 ± 0.45	-43.13 ± 4.1
2itz	-48.03 ± 3.1	-148.99 ± 16.4	159.64 ± 13.9	-6.22 ± 0.31	-43.60 ± 4.0
2j6m	-49.72 ± 3.3	-10.82 ± 2.1	21.93 ± 2.0	-6.09 ± 0.38	-44.71 ± 3.5
2itp	-51.58 ± 3.6	8.02 ± 3.1	2.11 ± 0.2	-6.41 ± 0.40	-47.86 ± 4.2
2itt	-49.40 ± 4.5	24.62 ± 5.5	-14.07 ± 4.5	-6.09 ± 0.60	-44.94 ± 5.6
5cav	-49.18 ± 3.2	-36.46 ± 7.5	52.72 ± 6.5	-5.88 ± 0.32	-38.81 ± 5.2
5cas	-55.05 ± 2.7	-38.65 ± 5.4	45.89 ± 4.3	-6.80 ± 0.21	-54.61 ± 4.0
5em8	-42.27 ± 4.3	-98.91 ± 19.2	108.91 ± 19.5	-5.17 ± 0.56	-37.45 ± 4.3
5em7	-52.92 ± 3.4	-123.02 ± 14.1	137.16 ± 14.3	-6.37 ± 0.36	-45.15 ± 4.0

Table S6. Binding free energy values for the protein-ligand systems during the first 25ns ($\Delta G_{pb\text{-}25ns}$) using the MMPBSA approach (in units of kcal/mol).

Systems	ΔE_{vdw}	ΔE_{ele}	$\Delta G_{ele,sol}$	$\Delta G_{npol,sol}$	$\Delta G_{pb\text{-}25ns}$
2ity	-45.45 ± 4.6	-128.25 ± 23.0	148.96 ± 23.7	-4.67 ± 0.31	-29.41 ± 6.7
2ito	-47.15 ± 4.2	-139.15 ± 18.9	162.82 ± 16.1	-4.70 ± 0.20	-28.19 ± 5.6
2itz	-47.43 ± 2.8	-131.58 ± 22.9	153.63 ± 19.0	-4.54 ± 0.17	-29.92 ± 5.2
2j6m	-48.80 ± 3.4	7.32 ± 1.8	20.22 ± 1.7	-4.97 ± 0.21	-26.22 ± 5.9
2itp	-52.44 ± 3.0	4.64 ± 1.6	25.67 ± 1.3	-5.20 ± 0.12	-27.32 ± 4.9
2itt	-49.26 ± 3.3	27.83 ± 2.1	-0.16 ± 0.01	-4.99 ± 0.23	-26.57 ± 4.0
5cav	-52.95 ± 2.9	-28.37 ± 9.4	56.19 ± 9.7	-4.78 ± 0.11	-29.92 ± 4.6
5cas	-52.36 ± 2.8	-34.76 ± 7.0	53.43 ± 5.5	-4.67 ± 0.07	-38.37 ± 3.9
5em8	-50.74 ± 3.2	-137.77 ± 26.4	160.09 ± 25.0	-4.90 ± 0.21	-33.33 ± 4.8
5em7	-49.52 ± 3.3	-132.42 ± 16.6	154.68 ± 17.0	-4.73 ± 0.20	-31.99 ± 3.9

Table S7. Binding free energy values for the protein-ligand systems during the last 50ns ($\Delta G_{pb\text{-}50ns}$) using the MMPBSA approach (in units of kcal/mol).

System	ΔE_{vdw}	ΔE_{ele}	$\Delta G_{ele,sol}$	$\Delta G_{npol,sol}$	$\Delta G_{pb\text{-}50ns}$
2ity	-48.02 ± 3.0	-162.68 ± 17.0	179.75 ± 15.0	-4.79 ± 0.14	-35.75 ± 5.5
2ito	-50.50 ± 3.7	-154.00 ± 21.0	179.52 ± 17.5	-4.90 ± 0.26	-29.88 ± 7.5
2itz	-48.02 ± 3.1	-148.99 ± 16.4	169.13 ± 13.0	-4.70 ± 0.17	-32.59 ± 5.2
2j6m	-49.72 ± 3.3	-10.82 ± 1.0	40.14 ± 22.7	-5.08 ± 0.20	-25.48 ± 4.7
2itp	-51.58 ± 3.6	8.02 ± 1.5	18.35 ± 1.5	-4.96 ± 0.30	-30.17 ± 5.0
2itt	-49.40 ± 4.5	24.62 ± 1.5	1.32 ± 0.15	-4.94 ± 0.34	-28.39 ± 5.5
5cav	-49.18 ± 3.2	-36.46 ± 7.5	59.16 ± 7.1	-4.63 ± 0.14	-31.11 ± 5.4

5cas	-55.05 ± 2.7	-38.65 ± 5.4	57.31 ± 5.5	-4.76 ± 0.09	-41.16 ± 3.9
5em8	-42.27 ± 4.3	-98.91 ± 19.3	117.89 ± 20.0	-4.44 ± 0.31	-27.75 ± 5.2
5em7	-52.92 ± 3.4	-123.02 ± 14.1	143.10 ± 15.0	-4.82 ± 0.20	-37.67 ± 3.8

Table S8. Binding free energy values for the protein-ligand systems during the first 25ns considering the entropic component ($\Delta G_{gbds-25ns}$) using the MMGBSA approach (in units of kcal/mol).

Systems	ΔE_{vdw}	ΔE_{ele}	$\Delta G_{ele,sol}$	$\Delta G_{npol,sol}$	$\Delta G_{rel-gb-5ns}$	-TDS	$\Delta G_{gbds-25ns}$
2ity	-45.03 ± 3.7	-139.8 ± 25.5	154.14 ± 23.4	-5.75 ± 0.63	-36.43 ± 4.6	-27.51 ± 8.9	-8.92
2ito	-50.31 ± 3.3	-136.7 ± 16.3	152.29 ± 16.4	-6.44 ± 0.43	-41.19 ± 3.1	-28.92 ± 4.2	-12.27
2itz	-51.04 ± 3.4	-136.5 ± 14.7	153.03 ± 13.6	-6.47 ± 0.43	-40.98 ± 4.0	-24.11 ± 4.7	-16.87
2j6m	-50.14 ± 3.0	24.36 ± 4.4	-11.41 ± 5.0	-6.13 ± 0.28	-43.32 ± 3.5	-17.82 ± 5.3	-25.5
2itp	-52.34 ± 3.3	16.11 ± 16.3	-5.34 ± 1.2	-6.39 ± 0.40	-47.96 ± 3.3	-11.83 ± 6.4	-36.13
2itt	-49.3 ± 2.7	18.93 ± 11.3	-7.65 ± 1.9	-5.99 ± 0.37	-44.08 ± 3.1	-21.07 ± 4.2	-23.01
5cav	-51.51 ± 2.5	-34.75 ± 6.2	52.37 ± 5.0	-6.21 ± 0.23	-40.11 ± 3.9	-20.90 ± 4.9	-19.21
5cas	-50.54 ± 3.1	-39.48 ± 6.0	47.89 ± 5.2	-6.46 ± 0.25	-48.59 ± 5.0	-20.79 ± 4.2	-27.8
5em8	-49.02 ± 4.3	-147.59 ± 18.9	159.17 ± 19.7	-6.27 ± 0.40	-43.72 ± 4.7	-18.47 ± 2.3	-25.25
5em7	-49.22 ± 3.3	-131.16 ± 19.7	144.23 ± 19.5	-6.10 ± 0.33	-42.25 ± 3.6	-16.29 ± 7.0	-25.96

Table S9. Binding free energy values for the protein-ligand systems during last 50 ns considering the entropic component ($\Delta G_{gbds\text{-}50ns}$) using the MMGBSA approach (in units of kcal/mol).

Systems	ΔE_{vdw}	ΔE_{ele}	$\Delta G_{ele,sol}$	$\Delta G_{npol,sol}$	$\Delta G_{rel\text{-}gb\text{-}50ns}$	-TDS	$\Delta G_{gbds\text{-}50ns}$
2ity	-48.02 ± 3.0	-162.68 ± 17.0	172.83 ± 15.9	-6.30 ± 0.38	-44.18 ± 4.3	-27.51 ± 8.9	-16.67
2ito	-50.50 ± 3.7	-154.00 ± 21.1	168.00 ± 18.5	-6.63 ± 0.45	-43.13 ± 4.1	-28.92 ± 4.2	-14.21
2itz	-48.03 ± 3.1	-148.99 ± 16.4	159.64 ± 13.9	-6.22 ± 0.31	-43.60 ± 4.0	-24.11 ± 4.7	-19.49
2j6m	-49.72 ± 3.3	-10.82 ± 2.1	21.93 ± 2.0	-6.09 ± 0.38	-44.71 ± 3.5	-17.82 ± 5.4	-26.89
2itp	-51.58 ± 3.6	8.02 ± 3.1	2.11 ± 0.2	-6.41 ± 0.40	-47.86 ± 4.2	-11.83 ± 6.4	-36.03
2itt	-49.40 ± 4.5	24.62 ± 5.5	-14.07 ± 4.5	-6.09 ± 0.60	-44.94 ± 5.6	-21.07 ± 4.2	-23.87
5cav	-49.18 ± 3.2	-36.46 ± 7.5	52.72 ± 6.5	-5.88 ± 0.32	-38.81 ± 5.2	-20.90 ± 4.9	-17.91
5cas	-55.05 ± 2.7	-38.65 ± 5.4	45.89 ± 4.3	-6.80 ± 0.21	-54.61 ± 4.0	-20.79 ± 4.2	-33.82
5em8	-42.27 ± 4.3	-98.91 ± 19.2	108.91 ± 19.5	-5.17 ± 0.56	-37.45 ± 4.3	-18.47 ± 2.3	-18.98
5em7	-52.92 ± 3.4	-123.02 ± 14.1	137.16 ± 14.3	-6.37 ± 0.36	-45.15 ± 4.0	-16.29 ± 7.0	-28.16

Table S10. Binding free energy values for the protein-ligand systems during the first 25ns considering the entropic component ($\Delta G_{pbds\text{-}25ns}$) using the MMPBSA approach (in units of kcal/mol).

System	ΔE_{vdw}	ΔE_{ele}	$\Delta G_{ele,sol}$	$\Delta G_{npol,sol}$	$\Delta G_{rel\text{-}pb\text{-}5ns}$	-TDS	$\Delta G_{pbds\text{-}25ns}$
2ity	-45.45 ± 4.6	-128.25 ± 23.0	148.96 ± 23.7	-4.67 ± 0.31	-29.41 ± 6.7	-27.51 ± 8.9	-1.9
2ito	-47.15 ± 4.2	-139.15 ± 18.9	162.82 ± 16.1	-4.70 ± 0.20	-28.19 ± 5.6	-28.92 ± 4.2	0.73
2itz	-47.43 ± 2.8	-131.58 ± 22.9	153.63 ± 19.0	-4.54 ± 0.17	-29.92 ± 5.2	-24.11 ± 4.7	-5.81
2j6m	-48.80 ± 3.4	7.32 ± 1.8	20.22 ± 1.7	-4.97 ± 0.21	-26.22 ± 5.9	-17.82 ± 5.3	-8.4
2itp	-52.44 ± 3.0	4.64 ± 1.6	25.67 ± 1.3	-5.20 ± 0.12	-27.32 ± 4.9	-11.83 ± 6.4	-15.49
2itt	-49.26 ± 3.3	27.83 ± 2.1	-0.16 ± 0.01	-4.99 ± 0.23	-26.57 ± 4.0	-21.07 ± 4.2	-5.5
5cav	-52.95 ± 2.9	-28.37 ± 9.4	56.19 ± 9.7	-4.78 ± 0.11	-29.92 ± 4.6	-20.90 ± 4.9	-9.02
5cas	-52.36 ± 2.8	-34.76 ± 7.0	53.43 ± 5.5	-4.67 ± 0.07	-38.37 ± 3.9	-20.79 ± 4.2	-17.58
5em8	-50.74 ± 3.2	-137.77 ± 26.4	160.09 ± 25.0	-4.90 ± 0.21	-33.33 ± 4.8	-18.47 ± 2.3	-14.86
5em7	-49.52 ± 3.3	-132.42 ± 16.6	154.68 ± 17.0	-4.73 ± 0.20	-31.99 ± 3.9	-16.29 ± 7.0	-15.7

Table S11. Binding free energy values for the protein-ligand systems during the last 50ns considering the entropic component ($\Delta G_{pbds-50ns}$) using the MMPBSA approach (in units of kcal/mol).

System	ΔE_{vdw}	ΔE_{ele}	$\Delta G_{ele,sol}$	$\Delta G_{npol,sol}$	$\Delta G_{rel-pb-50ns}$	-TDS	$\Delta G_{pbds-50ns}$
2ity	-48.02 ± 3.0	-162.68 ± 17.0	179.75 ± 15.0	-4.79 ± 0.14	-35.75 ± 5.5	-27.51 ± 8.9	-8.24
2ito	-50.50 ± 3.7	-154.00 ± 21.0	179.52 ± 17.5	-4.90 ± 0.26	-29.88 ± 7.5	-28.92 ± 4.2	-0.96
2itz	-48.02 ± 3.1	-148.99 ± 16.4	169.13 ± 13.0	-4.70 ± 0.17	-32.59 ± 5.2	-24.11 ± 4.7	-8.48
2j6m	-49.72 ± 3.3	-10.82 ± 1.0	40.14 ± 22.7	-5.08 ± 0.20	-25.48 ± 4.7	-17.82 ± 5.4	-7.66
2itp	-51.58 ± 3.6	8.02 ± 1.5	18.35 ± 1.5	-4.96 ± 0.30	-30.17 ± 5.0	-11.83 ± 6.4	-18.34
2itt	-49.40 ± 4.5	24.62 ± 1.5	1.32 ± 0.15	-4.94 ± 0.34	-28.39 ± 5.5	-21.07 ± 4.2	-7.32
5cav	-49.18 ± 3.2	-36.46 ± 7.5	59.16 ± 7.1	-4.63 ± 0.14	-31.11 ± 5.4	-20.90 ± 4.9	-10.21
5cas	-55.05 ± 2.7	-38.65 ± 5.4	57.31 ± 5.5	-4.76 ± 0.09	-41.16 ± 3.9	-20.79 ± 4.2	-20.37
5em8	-42.27 ± 4.3	-98.91 ± 19.3	117.89 ± 20.0	-4.44 ± 0.31	-27.75 ± 5.2	-18.47 ± 2.3	-9.28
5em7	-52.92 ± 3.4	-123.02 ± 14.1	143.10 ± 15.0	-4.82 ± 0.20	-37.67 ± 3.8	-16.29 ± 7.0	-21.38

Table S12. Binding free energy values (in kcal/mol) for the protein–ligand systems during the first

System	$\Delta G_{gb\text{-}25ns}$	$\Delta G_{gb\text{-}50ns}$	$\Delta G_{pb\text{-}25ns}$	$\Delta G_{pb\text{-}50ns}$	$\Delta G_{gbds\text{-}25ns}$	$\Delta G_{gbds\text{-}50ns}$	$\Delta G_{pbds\text{-}25ns}$	$\Delta G_{pbds\text{-}50ns}$
2ity	-36.43 ± 4.6	-44.18 ± 4.3	-29.41 ± 6.7	-35.75 ± 5.5	-8.92	-16.67	-1.9	-8.24
2ito	-41.19 ± 3.1	-43.13 ± 4.1	-28.19 ± 5.6	-29.88 ± 7.5	-12.27	-14.21	0.73	-0.96
2itz	-40.98 ± 4.0	-43.60 ± 4.0	-29.92 ± 5.2	-32.59 ± 5.2	-16.87	-19.49	-5.81	-8.48
2j6m	-43.32 ± 3.5	-44.71 ± 3.5	-26.22 ± 5.9	-25.48 ± 4.7	-25.5	-26.89	-8.4	-7.66
2itp	-47.96 ± 3.3	-47.86 ± 4.2	-27.32 ± 4.9	-30.17 ± 5.0	-36.13	-36.03	-15.49	-18.34
2itt	-44.08 ± 3.1	-44.94 ± 5.6	-26.57 ± 4.0	-28.39 ± 5.5	-23.01	-23.87	-5.5	-7.32
5cav	-40.11 ± 3.9	-38.81 ± 5.2	-29.92 ± 4.6	-31.11 ± 5.4	-19.21	-17.91	-9.02	-10.21
5cas	-48.59 ± 5.0	-54.61 ± 4.0	-38.37 ± 3.9	-41.16 ± 3.9	-27.8	-33.82	-17.58	-20.37
5em8	-43.72 ± 4.7	-37.45 ± 4.3	-33.33 ± 4.8	-27.75 ± 5.2	-25.25	-18.98	-14.86	-9.28
5em7	-42.25 ± 3.6	-45.15 ± 4.0	-31.99 ± 3.9	-37.67 ± 3.8	-25.96	-28.16	-15.7	-21.38

5 ns and the last 50 ns using the MMGBSA or MMPBSA approach.

Binding free energy values obtained during the first 25 or the last 50 ns using MMGBSA ($\Delta G_{gb\text{-}25ns}$ or $\Delta G_{gb\text{-}50ns}$) or MMPBSA ($\Delta G_{pb\text{-}25ns}$ or $\Delta G_{pb\text{-}50ns}$) approach. Values obtained during the first 25 ns or the last 50 ns using the MMGBSA ($\Delta G_{gbds\text{-}25ns}$ or $\Delta G_{gbds\text{-}50ns}$) or MMPBSA ($\Delta G_{pbds\text{-}25ns}$ or $\Delta G_{pbds\text{-}50ns}$) approach.

Table S13. $\Delta G_{docking}$ values (in kcal/mol) for the protein–ligand systems using two different docking programs. ΔG values obtained using MOE (ΔG_{MOE}), and SwissDock (ΔG_{SWDOCK}).

PDB entry	ΔG_{MOE}	ΔG_{SWDOCK}
4wa9	-7.0	-9.86
4twp	-9.14	-9.5
2ity	-7.24	-8.98
2ito	-7.02	-9.01
2itz	-6.27	-8.86
2j6m	-5.58	-10.35
2itp	-7.53	-8.74
2itt	-7.95	-9.74
5cav	-6.85	-8.28
5cas	-7.16	-9.6
5em8	-5.78	-9.9
5em7	-6.18	-10.77
4eor	-7.48	-8.55
4eok	-7.07	-8.0
1xh4	-12.97	-14.92
1xh9	-13.27	-15.85
3g0e	-6.97	-8.38
3g0f	-6.30	-8.03
5am6	-6.37	-9.08
5am7	-6.37	-8.03
4cli	-6.16	-8.40
4clj	-6.60	-8.99

References

1. Yun, C.-H.; Boggon, T. J.; Li, Y.; Woo, M. S.; Greulich, H.; Meyerson, M.; Eck, M. J. Structures of Lung Cancer-Derived EGFR Mutants and Inhibitor Complexes: Mechanism of Activation and Insights into Differential Inhibitor Sensitivity. *Cancer Cell* 2007, *11* (3), 217–227.
2. Heald, R.; Bowman, K. K.; Bryan, M. C.; Burdick, D.; Chan, B.; Chan, E.; Chen, Y.; Clausen, S.; Dominguez-Fernandez, B.; Eigenbrot, C.; Elliott, R.; Hanan, E. J.; Jackson, P.; Knight, J.; La, H.; Lainchbury, M.; Malek, S.; Mann, S.; Merchant, M.; Mortara, K.; Purkey, H.; Schaefer, G.; Schmidt, S.; Seward, E.; Sideris, S.; Shao, L.; Wang, S.; Yeap, K.; Yen, I.; Yu, C.; Heffron, T. P. Noncovalent Mutant Selective Epidermal Growth Factor Receptor Inhibitors: A Lead Optimization Case Study. *J. Med. Chem.* 2015, *58* (22), 8877–8895.
3. Bryan, M. C.; Burdick, D. J.; Chan, B. K.; Chen, Y.; Clausen, S.; Dotson, J.; Eigenbrot, C.; Elliott, R.; Hanan, E. J.; Heald, R.; Jackson, P.; La, H.; Lainchbury, M.; Malek, S.; Mann, S. E.; Purkey, H. E.; Schaefer, G.; Schmidt, S.; Seward, E.; Sideris, S.; Wang, S.; Yen, I.; Yu, C.; Heffron, T. P. Pyridones as Highly Selective, Noncovalent Inhibitors of T790M Double Mutants of EGFR. *ACS Med. Chem. Lett.* 2016, *7* (1), 100–104. https://doi.org/10.1021/acsmedchemlett.5b00428.
